# Principles of Endoscopic Surveillance of Extrapapillary Duodenal Lesions in Familial Adenomatous Polyposis: A 14-Year Single-Center Observation

**DOI:** 10.3390/cancers17152490

**Published:** 2025-07-28

**Authors:** Jarosław Cwaliński, Gabriela Kot, Wiktoria Grochowska, Katarzyna Budzyńska, Agnieszka Cwalińska, Jacek Paszkowski

**Affiliations:** 1 Department of General, Endocrinological Surgery and Gastroenterological Oncology, Poznan University of Medical Sciences, 61-701 Poznan, Poland; 2 Department of Infectious Diseases and Child Neurology, Poznan University of Medical Sciences, 61-701 Poznan, Poland

**Keywords:** duodenal polyposis, extrapapillary lesions, familial adenomatous polyposis, spigelman score, surveillance

## Abstract

**Background**: In patients with familial adenomatous polyposis (FAP), the duodenum is another high-risk region for malignancy after the large bowel. However, endoscopic and surgical management differs for papillary lesions and adenomas located in other parts of the duodenum. The aim of the study was to present the principles of the endoscopic surveillance of extrapapillary polyps based on a single-center 14-year observational study. **Methods**: The retrospective analysis was carried out in 2010–24 on a group of 45 people enrolled in endoscopic surveillance of the upper gastrointestinal tract due to FAP. The evaluation was aimed at detecting the malignant transformation of extrapapillary duodenal adenomas, with a radical removal of high-risk lesions. The severity of polyposis in the subsequent years of observation as well as the effectiveness of routine polypectomy on downstaging according to the Spiegelmann score were also assessed. **Results**: Invasive duodenal cancer was not detected in any case; however, high-grade dysplasia (HGD) was confirmed in five patients. The severity of polyposis and the number of polyps with HGD increased in following examinations, but routine polypectomy performed mainly during the 4th and 5th endoscopies allowed for a transient decrease in the Spiegelman score. Finally, progression of duodenal polyposis was observed in 18 patients, another 4 experienced regression (downstaging) and in 23 cases the stage of severity did not change. In addition, five patients were diagnosed with LST-G lesions, which were removed without recurrence. **Conclusions**: The patient’s age correlates with the severity of polyposis and the risk of malignancy, but routine endoscopic resections eliminate potentially invasive lesions and contribute to disease regression expressed by the Spiegelmann score. The radical endoscopic therapy of extrapapillary duodenal lesions limits the indications for surgical procedures.

## Simple Summary

Familial adenomatous polyposis (FAP) is a hereditary condition that causes numerous polyps in the digestive tract and significantly increases the risk of cancers. While most attention is given to the lesions in the colon, this study focuses on those located in the duodenum, outside the ampulla of Vater, which are often overlooked despite their cancerous potential. Over 14 years, we monitored 45 patients with FAP through regular endoscopic surveillance, aiming to detect and remove high-risk polyps before they could become malignant. Our findings indicate that consistent endoscopic surveillance and removal of these lesions may prevent invasive cancer and lead to a reduction in disease severity over time. This research highlights the importance of targeted, long-term monitoring of extrapapillary duodenal lesions and may also help with management strategies that could reduce the need for major surgeries among these patients.

## 1. Introduction

Familial adenomatous polyposis (FAP) is a genetically determined disease that affects both sexes with an incidence of 1/7500–1 in 10,000 individuals in the general population [1]. It typically manifests in adolescence as a result of an autosomal dominant inactivating mutation in the Adenomatous Polyposis Coli (APC) gene, located on chromosome 5 in the q21 region [1,2]. Although the detection of at least 100 adenomatous polyps in the large intestine is characteristic of classic FAP, an attenuated form of FAP with a reduced number of polyps is also recognized. Due to the hereditary nature of the disease and the high risk of carcinogenesis, the American National Comprehensive Cancer Network (NCCN) recommends genetic testing and the screening of first-degree relatives aged 10 to 15 years [3].

Both the number and size of polyps strongly correlate with the progression of dysplasia; hence, the risk of malignancy increases significantly in the subsequent decades of life. Long-term analysis has revealed that colorectal cancer develops in over 90% of patients after the age of 50, unless prophylactic surgical intervention is performed [2,4]. The treatment of choice is total colectomy, with optional preservation of the rectum, usually performed in the second decade of life and preceded by an annual colonoscopy starting from the age of 12 to 14 years [5].

In contrast, duodenal malignancy affects from 5 to 10% of patients, despite duodenal polyps occurring in up to 95% of individuals with FAP [6,7]. The location of duodenal adenomas is anatomically heterogeneous and may involve the ampulla of Vater, limiting the indications for definitive surgery [8]. Hence, endoscopy remains the primary diagnostic tool, and gastrointestinal (GI) surveillance contributes to the monitoring of polyposis as well as the removal of high-risk lesions. From the age of 25, gastroscopy should be performed regularly depending on the grades of the Spigelmann score (SS) [9]. However, one of the disadvantages of this classification lies in its assigning equal priority to all four evaluated parameters, potentially underestimating certain manifestations of the disease [10]. Particular atypical lesions, such as large polyps, laterally spreading tumors (LSTs), or adenomas involving the papilla of Vater, carry a higher predictive value of malignancy and require separate consideration [11].

Therefore, in our study, we present the goals of surveillance and the results of endoscopic therapy in FAP patients with extrapapillary duodenal lesions.

## 2. Materials and Methods

A group of 45 patients with extrapapillary duodenal lesions genetically determined by FAP qualified for endoscopic surveillance of the upper GI tract in the years 2010–2024. In each case, a baseline examination and at least one follow-up gastroscopy were performed at intervals of 1 to 4 years, depending on the initial SS and individual recommendations. A duodenal endoscopy was performed for diagnostic and therapeutic purposes to assess the number and diameter of polyps, as well as the degree of dysplasia. Patients with coexisting adenoma of the papilla of Vater as well as those who declined endoscopic follow-up or whose documentation was incomplete were excluded from the study.

### 2.1. Surveillance Endpoints

- Detection of invasive cancer and subsequent qualification for surgical resection.
- Excision of high-risk lesions, such as polyps with diameter >10 mm, foci of high-grade dysplasia, and LSTs.
- Evaluation of polyposis severity using the Spigelman score.

### 2.2. Diagnostic Criteria for Polyposis

The severity of polyposis was determined according to the SS, with mandatory assessment of the number and diameter of polyps during each endoscopy. Dysplasia was routinely evaluated using narrow-band imaging (NBI) and classified according to the Japan NBI Expert Team (JNET) classification adapted for the assessment of duodenal lesions. If the dysplasia of adenomas corresponded to JNET type 2A and the diameter of polyp did not exceed 5 mm biopsies were not routinely performed. In other cases, histopathological evaluation was obtained by biopsy or endoscopic resection and defined as low-grade dysplasia (LGD), high-grade dysplasia (HGD), or submucosal invasive cancer (SM). Moreover, lesions requiring individual observation and/or qualifying for endoscopic excision were morphologically classified using the Paris classification.

Endoscopic mucosal resection (EMR) was routinely performed for duodenal polyps of 10 mm or larger that did not exceed JNET grade 2A. The excision of smaller adenomas (5–10 mm) was also considered when lesion location or NBI characteristics suggested the benefit of the procedure.

Lesions with microscopic evidence of SM, or classified as grade 3 according to JNET, as well as post-polypectomy recurrences and suspected malignancies in imaging examinations were indicated for surgical treatment. Depending on the severity of neoplasia, transduodenal excision (TDE), pancreas-sparing duodenectomy (PSD), or pancreaticoduodenectomy (PD) were considered.

LSTs and lesions classified as grade 2B according to JNET classification were treated individually based on histopathological examination and imaging results, in particular MRI.

### 2.3. Statistical Analysis

Statistical analysis was performed using Statistica 12 software (Tibco Inc., Tulsa, OK, USA). Descriptive statistics were applied to measurable variables. Quantitative data were first evaluated for normal distribution using the Shapiro–Wilk test. Unless the data showed a normal distribution, they were reported as the median. A *p*-value of 0.05 or less was considered statistically significant.

## 3. Results

A total of 45 patients (27 women and 18 men), aged 23 to 68 years (mean age: 34 years) with confirmed duodenal polyposis were included in the retrospective analysis. Prophylactic total colectomy was performed in 42 patients. In the remaining three patients under 25 years of age, the procedure was temporarily postponed.

Histopathologically confirmed HGD was diagnosed in five patients. In one patient polypectomy of a lesion with recurrent focal HGD was performed twice during two consecutive endoscopies. No cases of invasive duodenal cancer were detected, therefore no patient required surgical resection.

**Figure 1:**
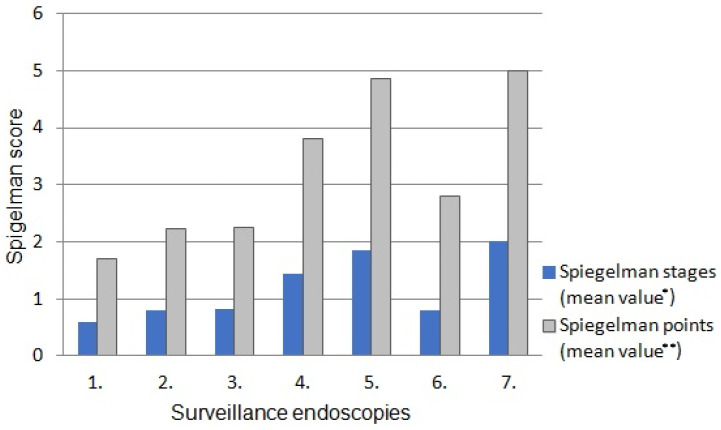
Severity of recurrence of adenomatous polyposis of the duodenum in successive endoscopic assessments, expressed in SS points and stages. * *p* = 0.0395, ** *p* = 0.0435.

**Figure 2:**
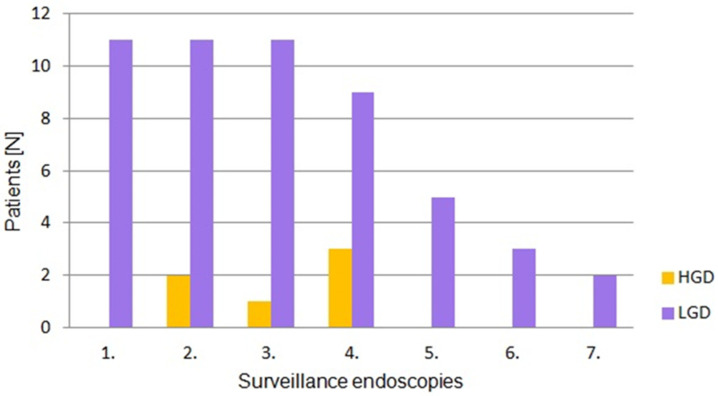
Grades of dysplasia according to histopathological evaluation in successive surveillance endoscopies. The specimens were obtained as a result of total polypectomy or EMR. HGD—High-Grade Dysplasia; LGD—Low-Grade Dysplasia.

**Figure 3:**
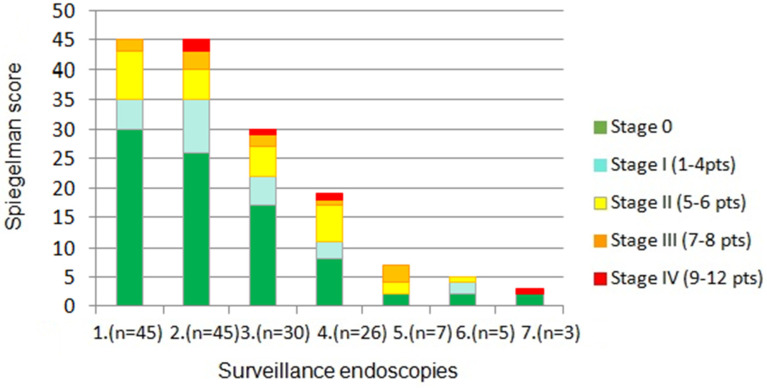
Severity adenomatous polyposis of the duodenum in subsequent endoscopic assessments, expressed in Spiegelman classification points and stages (*p* = 0.0265). Total number of examinations is given in brackets.

The severity of polyposis expressed in SS points and stages, as well as the rate of polyps with HGD, increased in the consecutive examinations, which correlated with the age of patients and duration of the disease (Figure 1 and Figure 2). This feature was mainly determined by a decrease in the percentage of cases with SS stage 0 during following examinations (Figure 3). However, regular polypectomy resulted in the elimination of high-risk lesions despite an extended surveillance period (Figure 2).

**Figure 4:**
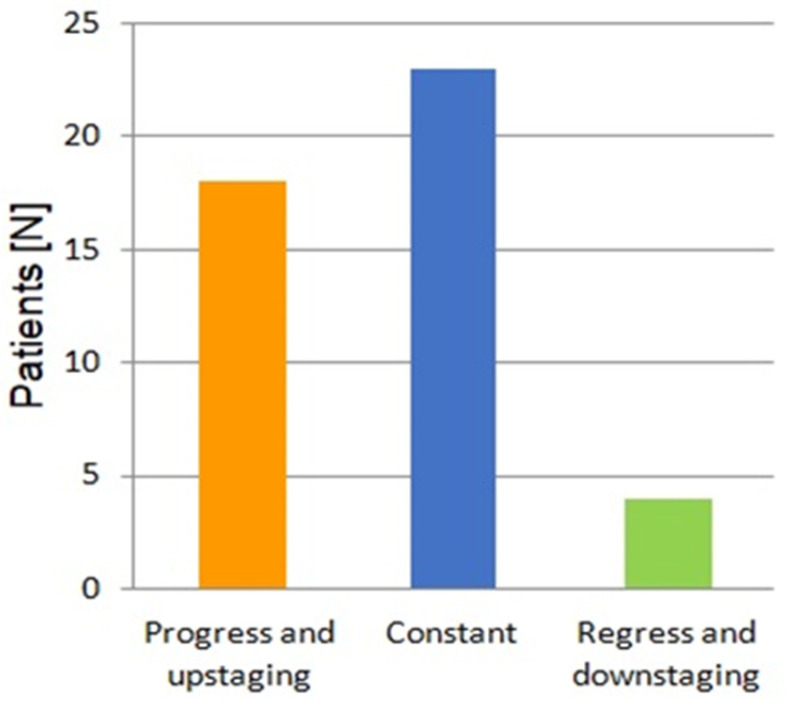
Summary of 14 years of endoscopic surveillance. Patients are divided into three groups based on severity of polyp progression compared to the initial examination. Analysis is based on SS points and stages.

Over the 14 years of the observation period, a progression of polyposis compared to the baseline was detected in 18 patients, while in more than half of the study group, the stage of the disease remained unchanged. A regression of duodenal polyposis with downstaging on the Spigelman scale was obtained in four patients (Figure 4). Furthermore, five patients with LST lesions underwent radical endoscopic resection without complications or recurrence in subsequent follow-up examinations.

## 4. Discussion

Effective endoscopic surveillance in patients with FAP is crucial for disease monitoring and the early detection of malignancy. Regular colon observation determines qualification for colectomy or proctocolectomy, followed by reconstructive surgery. Depending on the type of procedure, the remaining part of the rectum or intestinal pouch is subjected to further periodic endoluminal assessment in order to increase the cancer detection rate [12]. This strategy for managing the lower GI tract is well established and supported by guidelines significantly facilitating preoperative assessment and postoperative follow-up.

In contrast, strategies to minimize the risk of duodenal cancer are limited. Anatomical requirements, as well as restrictions of surgical management, underscore the importance of endoscopic surveillance of the duodenum in the group of patients with FAP [7,13]. International recommendations for upper GI surveillance rely on the SS, which assesses four parameters: the number, diameter, and histology of polyps, as well as the degree of dysplasia [14]. Based on the total score, patients can be assigned to one of five stages, each associated with a recommended follow-up interval. However, this period may vary depending on different guidelines (Table 1).

Although the management of the first stages of the SS is relatively consistent, recommendations differ in the more advanced stages of the disease. The ESGE, BSG, and joint EHTG-ESCP associations include the ampulla of Vater in the guidelines and incorporate ampullary polyps in the overall assessment of the duodenum [15,16,17,18] According to the most recent EHTG-ESCP update, if the papilla has an abnormal appearance, the suggested surveillance interval, based on the SS stage, should be shortened [18]. Alternatively, ESGE and BSG guidelines recommend a 3-year follow-up for polyps <10 mm and an annual endoscopy for lesions >10 mm [19,20].

As an alternative to the SS, some researchers rely strictly on selected high-risk features evaluated during follow-up. For example, Aelvoet et al. determine the timing of surveillance endoscopies based on the presence of incomplete resection, HGD, and the incidence of papillectomy. However, the time intervals remain consistent, following the paradigm of 5-, 3-, or 1-year intervals and 3–6 months, respectively [21].

Nevertheless, all of the aforementioned regimes identify endoscopy as the method of choice for the assessment of FAP progression and cancer avoidance. The prompt removal of all atypical lesions remains the priority in FAP patients, given that the duodenum is the second most common location for malignant transformation [22]. Research by Vasen et al. showed that regular endoscopic surveillance of the upper GI tract prolongs the expected survival of patients by approximately 7 months [23]. Simultaneously, Bülow et al. proved significant benefits of follow-up gastroscopy along with cancer prophylactic surgery, showing that patients with asymptomatic tumors detected through endoluminal visualization had a survival rate of 8 years, compared to 0.8 years for those whose cancer was identified based on symptoms alone [24].

The SS, introduced in 1989, became the gold standard for evaluating duodenal polyps in FAP patients and remains an essential tool for long-term follow-up planning [5,6,7,12,18]. Its main advantages include the scoring simplicity, availability of data in daily clinical practice, and high reliability, facilitating consistent stratification for surveillance across clinicians [25]. The study by Karstensen et al. demonstrated that the SS has an excellent reliability, with an intraclass correlation coefficient (ICC) of 0.95 for the overall scale, providing a reference for determining the risk of duodenal cancer [19]. According to Mannucci et al., the risk of malignancy is 16%, 3%, and 2% among patients assigned to SS stages IV, III, and II, respectively [26].

However, almost 40 years after its introduction, critical limitations of the Spigelman classification have been recognized. Non-invasive visualization available in the 1980s did not include modern techniques such as NBI and autofluorescence imaging (AFI); therefore, these advancements were not originally accounted for when determining surveillance intervals [27]. The results of follow-ups performed with modern endoscopic devices combined with endoluminal scoring systems and histological assessment indicate that the risk of cancer of both ampullary and extrapapillary lesions can be underestimated by the SS.

A study performed by Thiruvengadam et al. revealed that 53% of patients with duodenal cancer had never been classified in stage IV of the SS [28]. Similarly, Sourrouille et al. observed that among a group of 16 patients who developed adenocarcinoma, only 5 were classified as stage IV, and simultaneously at the fourth endoscopy, 30 patients had HGD polyps but only 13 of them were assigned to stage IV of the SS [29]. These findings highlight the potential limitations of the SS in fully capturing the severity of HGD lesions, which may not always correspond with the overall stage classification. The reason for this discrepancy may be the equal weighting of the criteria, whereas long-term analyses show a stronger correlation between larger polyp size (>10 mm) and the detection of HGD with an increased risk of malignancy. Another major disadvantage of the SS is the cumulative evaluation of papillary and extrapapillary lesions, which contradicts the observation that a large number of duodenal carcinomas are detected in the papilla, and adenomas of this area are usually characterized by more advanced dysplasia [28]. A summary the most essential features of the SS is presented in Table 2.

A breakthrough in the endoscopic assessment of gastrointestinal polyps was the introduction of narrow-band imaging (NBI) in the early 21st century. This technique utilizes specialized filters to narrow the spectrum of light absorbed by hemoglobin [30,31], enabling a detailed evaluation of tumor vascular patterns and mucosal architecture, thereby improving the detection of dysplasia and intramucosal carcinoma during endoscopy [32]. This advancement has led to the development of classification systems to categorize polyp types and grade dysplasia in lesions located in both the upper and lower gastrointestinal tract.

The Japan NBI Expert Team (JNET) classification was developed based on NBI and has become a valuable tool for distinguishing neoplastic from non-neoplastic lesions in the colon. The primary advantages of the JNET classification include high sensitivity, specificity, predictive value, and diagnostic accuracy. Moreover, it represents a favorable option for differential diagnosis that can be utilized by endoscopists at various levels of experience [33]. A common feature among patients with familial adenomatous polyposis (FAP) is the presence of adenomatous polyps, which require regular endoscopic surveillance due to the associated neoplastic risk. The NBI technique enables the precise and reproducible assessment of these lesions across all critical segments of the gastrointestinal tract, particularly the colon, duodenum, ileal pouch, and the ampulla of Vater. Furthermore, NBI-assisted endoscopy is widely available, easy to apply, and compatible with validated scoring systems for neoplasia risk stratification [34,35].

According to genetic predisposition the most common histologic type of duodenal polyps are tubular or villous adenomas. However, these are not the only lesions that may occur in the duodenum of FAP patients [6]. During long-term surveillance, such lesions as hyperplastic polyps, Brunner’s gland adenomas, hamartomas, and serrated or papillary adenomas also can be found in the duodenum [36]. Those lesions usually have a low malignancy risk; nevertheless they require careful histopathological differentiation from typical duodenal adenomas [37].

The management of duodenal lesions depends on their location relative to the papilla and on other criteria typically reported in the SS, such as polyp diameter, type of histology, and degree of dysplasia. Although adenomas are classified as non-ampullary if they do not invade the minor or major papilla, some polyps have no clear borders or partially involve the orifices of the biliary and/or pancreatic ducts [18,38]. The main goal of the procedure is complete resection while preserving histologically negative margins, which minimizes the rate of recurrences and prevents tumor progression. The technique and extent of polyp removal should also consider patients’ comorbidities, the risk of bleeding, the approximate depth of deep submucosal infiltration, and the experience of the endoscopist [39]. The non-invasive procedures used among the FAP population are similar to those used routinely in other groups of patients; however, they may differ for non-ampullary and ampullary lesions [34,40,41].

For the removal of small (<6 mm) non-ampullary lesions, cold snare polypectomy (CSP) is recommended [34], providing nearly 97% macroscopic radicality without coexisting complications such as bleeding or perforation [42]. An alternative solution is hot snare polypectomy (HSP), which includes lesion removal by wire loop with cauterization of the tissue by electric current. Although HSP has comparable efficacy to CSP, it is associated with a higher risk of delayed bleeding, favoring CSP as the preferred technique for removing small lesions in this location [43].

In contrast EMR remains the technique of choice for the treatment of non-ampullary polyps with a diameter of 10 mm or larger and includes a submucosal liquid injection followed by the snaring and transection of the polyp with an electric current [34,44]. This procedure achieves a high rate of complete resection (>90%), but is related to a higher risk of complications. Adverse effects occur in about 23% of patients, with the most common being delayed bleeding [45,46]. In order to increase EMR efficacy and safety, it is possible to use two modifications such as underwater EMR (UEMR) or cap-assisted EMR (EMR-C). UEMR involves the infusion of water into the duodenum until its lumen is completely filled, and subsequent lesion resection, without the need for a submucosal injection [47], whereas EMR-C relies on pulling up the lesion into a special cap before excision [48].

Adenomas with advanced HGD that display a high risk of deep penetration as well as lesions with scarring or fibrotic tissue should be qualified for endoscopic submucosal dissection (ESD) [34]. By elevating the lesion from the muscle layer followed by its removal with a needle knife, ESD ensures high radicality, including for foci of T1 cancer. However, ESD requires an experienced specialist and a well-equipped endoscopic center; therefore, it carries a high risk of complications [49].

LSTs, despite their relatively large dimensions, rarely undergo neoplastic transformation [50,51]. Apart from the Paris classification, the dedicated LST classification defined by Kudo et al. is also used for clinical stratification [52]. Unfortunately, the treatment of these lesions in patients with FAP is solely based on individual experience as long-term data regarding the risk of cancer transformation are limited. However, recent studies support the validity of LST resection using endoscopic techniques, such as EMR or ESD [53,54,55].

To summarize our surveillance strategy, we intentionally excluded adenomas of the papilla of Vater, recognizing that the diagnosis and therapy of these lesions differs from the management of other parts of duodenum. Our rationale for this approach was based on the following reasons: (1) both our findings and the EHTG-ESCP guidelines suggest that the use of the SS to describe lesions of the papilla of Vater may underestimate the risk of developing an ampullary adenocarcinoma and delay the appropriate treatment; (2) there is a significant difference in the endoscopic and surgical techniques used for the removal of ampullary polyps and lesions located in other duodenal regions; (3) the severity of complications following removal attempts of lesions in these two anatomical locations differs significantly; and (4) the recurrence rate of polyposis after the primary radical excision of adenomas located in the papilla of Vater is higher than for other duodenal lesions. In our study, patients with isolated adenomas in the papilla of Vater—i.e., without coexisting polyps in other parts of the duodenum—often corresponded to a low final score on the SS. However, the cumulative risk of malignant transformation, based predominantly on the polyp diameter, type, and grade of dysplasia, remains significant. Hence, we advocate for the separate endoscopic surveillance of papillary lesions and other parts of the duodenum, with mandatory NBI evaluation of polyps as a method to detect highly dysplastic lesions.

The use of the SS in the routine assessment of FAP patients who qualify for endoscopic surveillance requires experience and the reasonable interpretation of endoscopic findings. This applies not only to the accurate evaluation of the size and number of polyps, but especially to the decision-making regarding the need for an endoscopic biopsy, as the routinely performed biopsy can contribute to a falsely elevated SS. For example, the presence of numerous (>20) but small (<5 mm) polyps results in a final score of four points, which corresponds to stage I of the SS. Performing a biopsy, although it is not obligatory in this scenario, increases the score by two points, (one point for histology and one point for dysplasia), which ultimately advances the SS to stage II.

## 5. Limitations

In our study, we primarily focused on the practical aspects of duodenal endoscopy in patients with FAP. We considered the benefits and limitations of routinely used scales and the possibility of treatment with new endoscopic techniques. We intentionally applied the JNET classification for duodenal polyps’ assessment, as it provides a reliable correlation between the NBI-enhanced mucosal visualization and the histological grade of dysplasia. This approach is further supported by the fact that the vast majority of duodenal lesions in this patient group are adenomas, for which the JNET classification demonstrates high predictive accuracy [56]. We did not analyze in detail the effect of patients’ age on the severity of polyposis and the results of surveillance. As our population was too small and the patients enrolling in the endoscopic follow-up varied in age as well as the total number of performed endoscopies, we considered that this variable might confound the final results. Instead, we assessed the evolution of polyposis in increasingly older patients over successive years of surveillance.

## 6. Conclusions

This single-center study highlights the clinical importance of targeted endoscopic surveillance for extrapapillary lesions among patients with FAP. Our findings confirm that the severity of duodenal polyposis has a tendency to increase with age and disease duration; however, regular endoscopic evaluation and prompt polypectomy significantly reduce the risk of malignant tumor progression. Furthermore, the study underscores the limitations of the SS, particularly its inability to distinguish between papillary and extrapapillary lesions, suggesting the need for a more nuanced approach to risk stratification.

By focusing on extrapapillary lesions and excluding ampullary adenomas from standard scoring, we advocate for individualized management strategies that optimize patient outcomes, while reducing unnecessary surgical interventions.

## Figures and Tables

**Table 1:** Surveillance interval according to European Society of Gastrointestinal Endoscopy (ESGE); British Society of Gastroenterology (BSG); American Society of Gastrointestinal Endoscopy (ASGE); European Hereditary Tumor Group (EHTG); European Society of Coloproctology (ESCP).

Spigelman Stages	Surveillance Interval			
	ESGE, 2019r. [15]	BSG, 2020r. [16]	ASGE, 2020r. [17]	Joint EHTG-ESCP Revision; 2024 r. [18]
0	5 years	5 years	5 years	3–5 years
I	5 years	5 years	5 years	2–3 years or 2 years *
II	3 years	3 years	3 years	2 years or 1 year *
III	1 year	1 year	6–12 months	1 year or 6–12 months *
IV	6 months	6–12 months	3–6 months	6–12 months

* Interval depends on appearance of papilla.

**Table 2:** Advantages and disadvantages of the Spigelman Scale.

Advantages	Disadvantages
Guideline integration	Underestimation of malignancy risk
Simplicity and accessibility	Equal weighting of criteria
High reliability	Exclusion of papillary pathology
Association with cancer risk	Advancement in diagnostics not yet integrated

## Data Availability

The raw data supporting the conclusions of this article will be made available by the authors on request.

## Abbreviations

The following abbreviations are used in this manuscript:

FAP	Familial Adenomatous Polyposis
HGD	High-Grade Dysplasia
APC	Adenomatous Polyposis Coli
NCCN	National Comprehensive Cancer Network
GI	Gastrointestinal
SS	Spigelman Score
LSTs	Laterally Spreading Tumors
JNET	Japan NBI Expert Team
LGD	Low-Grade Dysplasia
SM	Submucosal Invasive Cancer
EMR	Endoscopic Mucosal Resection
TDE	Transduodenal Excision
PSD	Pancreas-Sparing Duodenectomy
PD	Pancreaticoduodenectomy
ESGE	European Society of Gastrointestinal Endoscopy
BSG	British Society of Gastroenterology
ASGE	American Society of Gastrointestinal Endoscopy
EHTG	European Hereditary Tumor Group
ESCP	European Society of Coloproctology
ICC	Intraclass Correlation Coefficient
AFI	Autofluorescence Imaging
CSP	Cold Snare Polypectomy
HSP	Hot Snare Polypectomy
UEMR	Underwater Endoscopic Mucosal Resection
EMR-C	Cap-Assisted Endoscopic Mucosal Resection
ESD	Endoscopic Submucosal Dissection
